# The Different Inhibition of Return (IOR) Effects of Emergency Managerial Experts and Novices: An Event-Related Potentials Study

**DOI:** 10.3389/fnbeh.2017.00090

**Published:** 2017-05-22

**Authors:** Rong Cao, Lü Wu, Shuzhen Wang

**Affiliations:** School of Public Management, Northwest UniversityXi’an, China

**Keywords:** inhibition of return, emergency managerial expert, novice, emergency pictures, ERPs

## Abstract

Inhibition of return (IOR) is an important effect of attention. However, the IOR of emergency managerial experts is unknown. By employing emergency and natural scene pictures in expert-novice paradigm, the present study explored the neural activity underlying the IOR effects for emergency managerial experts and novices. In behavioral results, there were no differences of IOR effects between novices and emergency managerial experts, while the event-related potentials (ERPs) results were different between novices and experts. In Experiment 1 (novice group), ERPs results showed no any IOR was robust at both stimulus-onset asynchrony (SOA) of 200 ms and 400 ms. In Experiment 2 (expert group), ERPs results showed an enhanced N2 at SOA of 200 ms and attenuated P3 at cued location in the right parietal lobe and adjacent brain regions than uncued location at SOA of 200 ms. The findings of the two experiments showed that, relative to the novices, IOR for the emergency managerial experts was robust, and dominated in the right parietal lobe and adjacent brain regions, suggesting more flexible attentional processing and higher visual search efficiency of the emergency managerial experts. The findings indicate that the P3, possible N2, over the right parietal lobe and adjacent brain regions are the biological indicators for IOR elicited by post-cued emergency pictures for emergency managerial experts.

## Introduction

Attention orients directly the most relevant stimuli and ignores irrelevant stimuli to given targets, and is thought to play an important role in human information processing (Eriksen and Hoffman, 1973; Jonides, 1976; Mountcastle, 1978; Posner, 1980; Wurtz et al., 1980; Hawkins et al., 1988; Remington et al., 1992). Inhibition of return (IOR), one of attentional effects, which is closely related to the ability to prevent orienting back to the previously attended locations (Posner and Cohen, 1984; Maylor, 1985; Rafal et al., 1989; Pratt, 1995; Cheal et al., 1998; Cheal and Chastain, 1999), was proposed by Posner and Cohen (1984). Assuming that the stimulus onset asynchrony (SOA) between a cue and a target is longer than 300 ms, the reaction times (RTs) are slower for the target presenting at the same location as a cue than that at the different location as a cue. IOR has already been researched in different kinds of experimental situations, such as location, color, shape and so on (Spence et al., 2000; Francis and Milliken, 2003; Lucia et al., 2004). Emergency managerial experts play an important role in the process of emergency management (Sayegh et al., 2004), and the IOR effects of emergency managerial experts and novices are hardly known. For IOR reflects the selection of information and visual searching efficiency (Klein, 2000; Macinnes and Klein, 2003; Wang and Klein, 2010), the aim of the present study is to explore IOR effects for emergency managerial experts and novices.

Previous studies have suggested that it takes at least a decade to be an expert in a specific domain (Bryan and Harter, 1897, 1899), such as the chess experts, experienced athletes and aviators. To test the individual differences in a specific domain, the expert-novice paradigm is employed by previous studies. Chess experts could quickly search for a similar position in the extraction of new information, and the activities in cingulate, orbitofrontal cortex, and right temporal lobe cortex of them were more active compared to the novices in the visual processing of chess (Chase and Simon, 1973a,b; Krawczyk et al., 2011).

To date, the issue for neural substrate of IOR effects has been examined by prior event-related potentials (ERPs) studies, and the ERPs technique has been proved to be a useful approach to examining the neural mechanism underlying the IOR for its high temporal resolution. N1, N2 and P3 are components of ERPs. N1 is a negative-going evoked potential, peaking between 150 and 200 ms after the onset of a stimulus in posterior scalp and distributing over the parietal and occipital lobe, which reflects discrimination process (Vogel and Luch, 2000; Hopt et al., 2002). N2 is also a negative-going waveform, peaking between 200–300 ms post-stimulus. Posterior distributions over the parietal lobe in visual attention have been reported in visual attention paradigms (Folstein and Van Petten, 2008). P3 is a waveform with positive amplitude, peaking at around 300 ms and the highest amplitudes typically distributing over parietal brain areas. P3 requires and reflects attention process (Kok, 2001). Previous ERPs studies have provided evidence for the cognitive mechanism underlying IOR. Some studies found that a smaller N1 amplitude were evoked by targets at cued locations than uncued locations (Prime and Ward, 2004; Chica and Lupiáñez, 2009; Prime and Jolicoeur, 2009). On the contrary, a larger N1 amplitude was found at cued locations than uncued locations (McDonald et al., 1999). Other studies suggested no IOR effect on N1 was found at cued locations and uncued locations (Hopfinger and Mangun, 2001; Satel et al., 2012). One study by Wright et al. (2013) suggested that experts showed that targets elicited an enhanced posterior N2 which started as early as 240 ms, relative to novices. Like the findings of N1, previous studies of P3 showed variable results. Some studies found smaller P3 amplitudes for targets at cued locations than uncued locations (Eimer, 1994). However, others found opposite patterns of P3 amplitude at cued and uncued locations (McDonald et al., 1999). Besides, the IOR effect on P3 was not found at cued and uncued locations even though the IOR effect was obtained in behavioral results (Hopfinger and Mangun, 2001; Zhang et al., 2012). Additionally, Wright et al. (2013) suggested that, relative to novices, experts showed an enhanced P3 for targets. It is obvious that the findings on the neural mechanism underlying IOR is inconsistent.

Although prior studies have examined cognitive and neural differences between experts and novices in some domains, the neural mechanisms underlying IOR for the managerial experts and novices remain unclear. Moreover, the brain activities underlying IOR effects for emergency managerial experts and novices are totally lack of understanding. In context of crisis, the rapid process is essential for emergency managerial experts. Because of expert judgments being very rapid (Gobet and Simon, 2000), we predicted that the expertise of emergency managerial experts could also be demonstrated in the neural activities. To investigate this issue, we selected the real emergency pictures as experimental stimuli vs. natural scene pictures for the present study in order to being ecological valid, and expected the IOR effects between emergency managerial experts and novices could be different in behavioral RTs, as well as in amplitudes of N1, N2 and P3 components.

## Experiment 1

### Method

#### Participants

Twenty-five volunteers of novices (3 females and 22 males, mean age 26.24 years, range from 20 years to 35 years old) participated in Experiment 1, consisting of general college students from the Northwest university and civil servants. They did not perform in related work of emergency management. Only one of them was left-hand. All participants reported normal or corrected-to-normal vision and had no history of current or past neurological or psychiatric illness and took no medications known to affect the central nervous system. Furthermore, written informed consent was obtained from all the participants before the beginning of experiment, and the experiment was approved by the Departmental Research Ethics Committee.

#### Stimuli and Experimental Design

The stimuli of the present experiment consist of two kinds of pictures: emergency and landscape pictures. Two-hundred and fifty-six emergency pictures contain four types of crisis events according to the Emergency Response Law of the People’s Republic of China, including natural disaster pictures (such as earthquake, flood pictures etc.), accidental disaster pictures (such as air crash, explosion pictures etc.), public health event pictures (such as SARS epidemic, pandemic influenza pictures etc.), and social security event pictures (such as attack terrorism, air raid pictures etc.). Two-hundred and fifty-six landscape pictures, which matched with the emergency pictures in perceptual features, such as size, pixel and so on, were selected as stimuli. Because pictures could automatically attracts individuals’ attention when a cue appears in visual field (Gutiérrez-Domínguez et al., 2014), to achieve the counter-balanced field of vision, the stimuli-presenting mode of bilateral vision was adopted in this experiment, i.e., one emergency picture and one landscape picture respectively presented each side of fixation, but only emergency pictures were analyzed when calculating data. A 2 (SOA: 200 ms vs. 400 ms) × 2 (Cue validity: cued vs. uncued) within-subjects design was employed. All statistical results were adjusted by employing the method of Greenhouse-Geisser correction.

#### Procedure

There was instruction before the beginning of the experiment. Participants were seated in a dimly illuminated, sound attenuated room, faced with a computer screen at a distance of 75 cm. The computer monitor screen (CRT: 1024 × 768) was placed and all stimuli were presented within 6° of visual angle in two sides of the screen. The background color of the monitor was black. At the start of each trial, a small white cross lasting 750 ms as to fixation was presented on the computer screen. Participants were instructed to maintain their eyes on central fixation during the task performance. After this, two kinds of pictures (emergency pictures and landscape pictures) would be presented at the same time, and the duration was 200 ms. Following the pictures, two intervals of 0 ms or 200 ms (SOA = 200 ms or 400 ms) were randomly presented. When the intervals disappeared, a rectangle or a triangle as a target would appear at random. Participants were asked to responsed to the target (rectangle or triangle) irrespective of its location by pressing one key as quickly and accurately as possible. If the target was a rectangle, press “F” button; otherwise, press “J” button. The assignment of response key to each condition was counter-balanced across participants. A target was not disappeared until a response was made or for a maximum of 2000 ms. The inter-trial-interval (ITI) was a empty screen that randomly varied between 500 ms and 800 ms.

**Figure 1:**
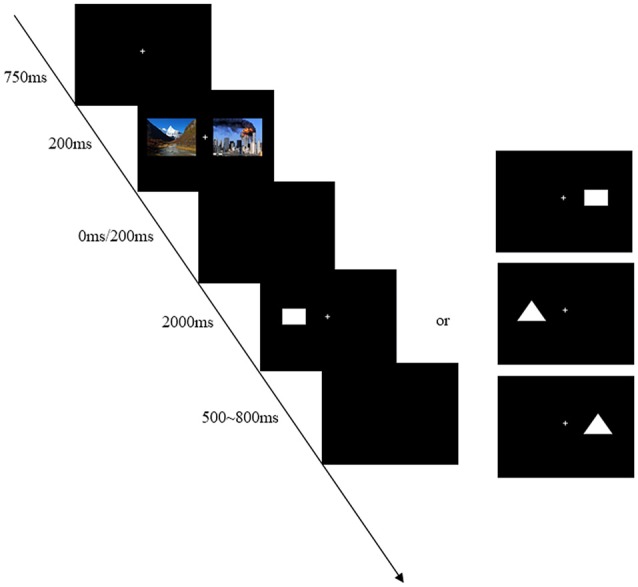
**An example of timing and sequence of stimuli**. First, the stimuli-presenting mode of bilateral vision was adopted in this experiment in order to avoid the automatic attraction of attention for the unilateral vision. Second, two kinds of pictures (emergency pictures and landscape pictures) would be presented at the same time so as to avoid the sequence effect. For instance, one landscape picture was presented on the left side and one emergency picture was presented on the right side in Figure 1. The trials with different locations were embedded within each block. Finally, the experiment was divided into two kinds of pressing buttons to be counter-balanced across participants, including “F–J” and “J–F” response patterns.

The whole experiment was divided into two sections, practice and experiment. There were 10 trials in practice section, and the task would cycle until the participants understood the experiment procedure. The experiment section contained 512 trials and was divided into four blocks of 128 trials. The different conditions (SOA = 200 ms or 400 ms) were randomly intermixed in each block. The experiment lasted about 25 min. Short breaks (the lengths of breaks were up to each participant) were allowed between blocks. The typical trial is shown in Figure 1.

### Recording and Analysis

#### Behavioral Recording and Data Analyses

The RTs and error rates of target stimuli in all conditions were on-line recorded by E-Prime software (Version 2.0). Only RTs of correct responses were used for data analysis. We eliminated the trials in which RTs were less than 100 ms or greater than 1000 ms (Gouzoulis-Mayfrank et al., 2006), and removed the data of participants whose RTs were above or below three standard deviations (SDs) from the mean of each condition (Vanselst and Jolicoeur, 1994).

#### EEG Recording and Data Analyses

The electroencephalogram (EEG) activity was continuously recorded and off-line data analyses were employed with Neurolab system by a set of 64 scalp Ag/AgCI electrodes placed according to the 10/20 international system. The tip of nose was used as reference during recording, and was re-referenced to M1 and M2 off-line. Vertical and horizontal electrooculogram (HEOG and VEOG) were recorded through electrodes placed on the bilateral external canthi and the left infraorbital and supraorbital areas. Electrode impedances were kept below 5 kΩ. The sampling rate was 500 Hz/channel. The EEG signals of each participant were continuously recorded by amplifier system and filtered online with a 0.05–100 Hz band pass. The data of ERPs were baseline corrected and segmented in epochs of 1000 ms post stimulus and 200 ms prior to stimulus onset. Any electrodes with amplitudes beyond ±100 μυ were excluded from averaging, and the averaged ERPs were low-pass filtered at 30 Hz (24 dB/octave).

#### Results

In the present experiment, five participants were eliminated according to the criteria of exclusion in behavioral and ERPs data analyses mentioned above. The remaining participants were 20 novices in crisis management domain.

#### Behavioral Results

Mean RTs and error rates for all conditions are calculated for each participant and submitted to a 2 (SOA: 200 ms vs. 400 ms) × 2 (Cue validity: cued vs. uncued) repeated measures analysis of ANOVA (see Table 1). The main effect of SOA was significant for RTs, *F*_(1,19)_ = 44.994 (*p* < 0.001), partial *η*^2^ = 0.703, suggesting that RTs at SOA of 200 ms (534.94 ± 68.77 ms) were much longer than those at SOA of 400 ms (512.42 ± 73.72 ms). The main effect of Cue validity was significant as well, *F*_(1,19)_ = 8.627, *p* = 0.008 (*p* < 0.01), partial *η*^2^ = 0.312, indicating that RTs in cued trials (526.58 ± 74.09 ms) were much longer than those in uncued trials (520.78 ± 70.13 ms). The main effect of Cue validity showed that there were IOR effects at SOA of 200 ms and 400 ms. The interaction between SOA and Cue validity was not significant, *p* = 0.757 (*p* > 0.05). For the error rates, no main effects and interaction were found (*p*s > 0.05).

**Table 1:** **Mean reaction times (RTs) (ms) and error rates (%) of the novices**.

SOA (ms)	Cue validity (M ± SD)	Mean RTs (M ± SD)	Error rates (%)
200	cued	537.52 ± 71.32	1.52 ± 1.39
	uncued	532.36 ± 67.89	1.60 ± 2.25
400	cued	515.63 ± 77.15	1.95 ± 1.89
	uncued	509.20 ± 72.21	1.68 ± 1.44

*Difference was equal to the subtraction mean RTs with cued condition minus uncued condition*.

#### Event-Related Potentials (ERPs) Results

**Figure 2:**
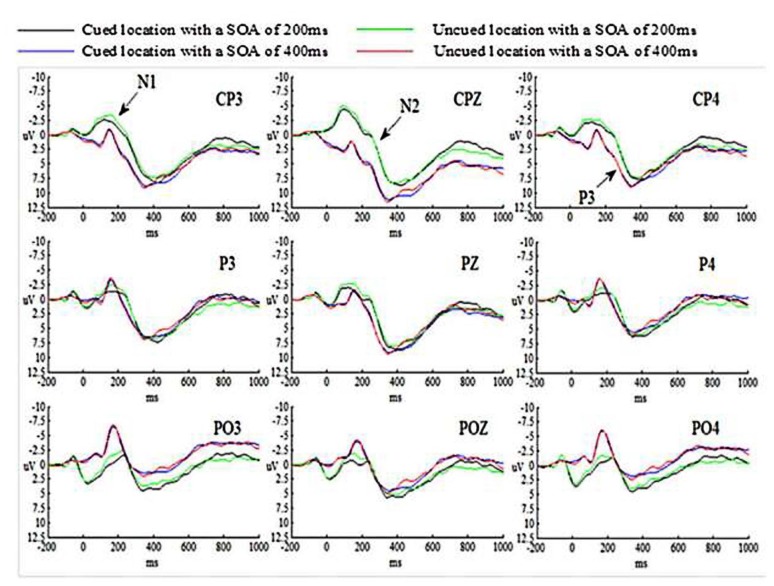
**Grand-averaged event-related potential (ERP) waveforms for the novices in Experiment 1**.

The grand-average ERP waveforms for all conditions are presented in Figure 2.

##### N1 component

N1 amplitude was measured by mean detection within a time window from 110 ms to 180 ms at CP3, CPZ, CP4, P3, PZ, P4, PO3, POZ and PO4 electrode sites. The N1 amplitude for all conditions were analyzed by employed an ANOVA of 2 (SOA: 200 ms vs. 400 ms) × 2 (Cue validity: cued vs. uncued) × 3 (Hem: left vs. central vs. right hemisphere) × 3 (Site: CP3/CPZ/CP4 vs. P3/PZ/P4 vs. PO3/POZ/PO4) repeated measures analysis.

The main effect of SOA was significant (*p* < 0.05), suggesting that N1 amplitude evoked by targets at SOA of 200 ms was much larger than those at SOA of 400 ms. The main effects of Cue validity and Hem were not significant (*p*s > 0.05). The main effect of Site was significant (*p* < 0.01), indicating that N1 amplitude evoked by targets at central-parietal cortex was smaller than those at parietal cortex, *p* = 0.011 (*p* < 0.05) and at parietal-occipital cortex, *p* = 0.001 (*p* < 0.01). N1 amplitude evoked by targets at parietal cortex was smaller than those at parietal-occipital cortex as well, *p* = 0.001 (*p* < 0.01). All interactions were not significant (*p*s > 0.05).

##### N2 component

N2 amplitude was calculated as the mean amplitude within a time window from 210 ms to 290 ms at the same electrode sites as in N1. The experimental design for N2 was identical with that for N1.

The main effect of SOA was significant (*p* < 0.01), suggesting that N2 amplitude evoked by targets at SOA of 200 ms was much larger than those at SOA of 400 ms. Both main effects of Cue validity and Hem were not significant (*p*s > 0.05). The main effect of Site was significant (*p* < 0.01), indicating that N2 amplitude evoked by targets at central-parietal cortex was smaller than those at parietal cortex and at parietal-occipital cortex (*p*s < 0.001). N2 amplitude evoked by targets at parietal cortex was smaller than those at parietal-occipital cortex as well, *p* = 0.008 (*p* < 0.01). All interactions were not significant (*p*s > 0.05).

##### P3 component

P3 amplitude was calculated as the mean amplitude within a time window from 290 ms to 520 ms at the same electrode sites as in N1. The experimental design for P3 was identical with that for N1.

The main effect of SOA was significant (*p* < 0.05), suggesting that P3 amplitude evoked by targets at SOA of 200 ms was smaller than those at SOA of 400 ms. The main effects of Cue validity and Hem were not significant (*p*s > 0.05). The main effect of Site was significant (*p* < 0.01), indicating that P3 amplitude evoked by targets at central-parietal cortex was much larger than those at parietal cortex (*p* < 0.001) and at parietal-occipital cortex, *p* = 0.006 (*p* < 0.01). P3 amplitude evoked by targets at parietal cortex and parietal-occipital cortex was not significant, *p* = 0.290 (*p* > 0.05). All interactions were not significant (*p*s > 0.05).

## Experiment 2

### Method

Experiment 1 revealed that there was an IOR effect for emergency pictures at SOA of 400 ms for novices. Using the same task of Experiment 1, the unique feature in Experiment 2 was the participants consisting of emergency managerial experts, thus all methods except participants in Experiment 2 were as the same as Experiment 1.

### Participants

Twenty-five volunteers of emergency managerial experts (1 female and 24 males, mean age: 44.08 years, range from 33 years to 58 years old; mean working years: 10.8, range from 2 to 34 years) participated in Experiment 2 and one of them was a left-hand. All of them were civil servants and engaged in emergency management. Other characteristics of participants were identical with the participants in Experiment 1. Written informed consents were obtained from all the participants before the experiment. The experiment was approved by the Departmental Research Ethics Committee. Five participants were eliminated according to the criteria of rejection in behavioral and ERPs data analyses mentioned above. Therefore, the remaining participants were 20 emergency managerial experts.

### Results

#### Behavioral Results

Mean RTs and error rates for all conditions are presented in Table 2. The main effect of SOA was significant for RTs, *F*_(1,19)_ = 100.947 (*p* < 0.001), partial *η*^2^ = 0.842, suggesting that RTs at SOA of 200 ms (581.89 ± 48.43 ms) were much longer than those at SOA of 400 ms (546.63 ± 53.98 ms). The main effect of Cue validity was significant as well, *F*_(1,19)_ = 4.792; *p* = 0.041 (*p* < 0.05), partial *η*^2^ = 0.201, indicating that RTs in cued trails (566.38 ± 55.60 ms) were much longer than those in uncued trials (562.15 ± 52.88 ms). The main effect of Cue validity showed that there were IOR effects at SOA of 200 ms and 400 ms. The interaction between SOA and Cue validity was also significant, *F*_(1,19)_ = 8.069; *p* = 0.010 (*p* < 0.05), partial *η*^2^ = 0.298. The simple effect on further test for comparison between cued and uncued condition at SOA of 200 ms showed that a significant 9.78 ms increase in RTs in cued trials, compared with the RTs in uncued trails, *p* = 0.015 (*p* < 0.05), but not for the SOA of 400 ms, *p* = 0.645 (*p* > 0.05), suggesting that there was a significant IOR effect for emergency pictures at SOA of 200 ms for emergency managerial experts. For error rates, no significant main effects and interaction were found (*p*s > 0.05).

**Table 2:** **Mean RTs (ms) and error rates (%) of the emergency managerial experts**.

SOA (ms)	Cue validity (M ± SD)	Mean RTs (M ± SD)	Error rates (%)
200	cued	586.80 ± 49.58	1.44 ± 1.55
	uncued	577.01 ± 48.03	1.41 ± 1.28
400	cued	545.98 ± 54.88	1.56 ± 1.48
	uncued	547.29 ± 54.48	1.56 ± 2.01

*Difference was equal to the subtraction mean RTs with cued condition minus uncued condition*.

#### Event-Related Potentials (ERPs) Results

**Figure 3:**
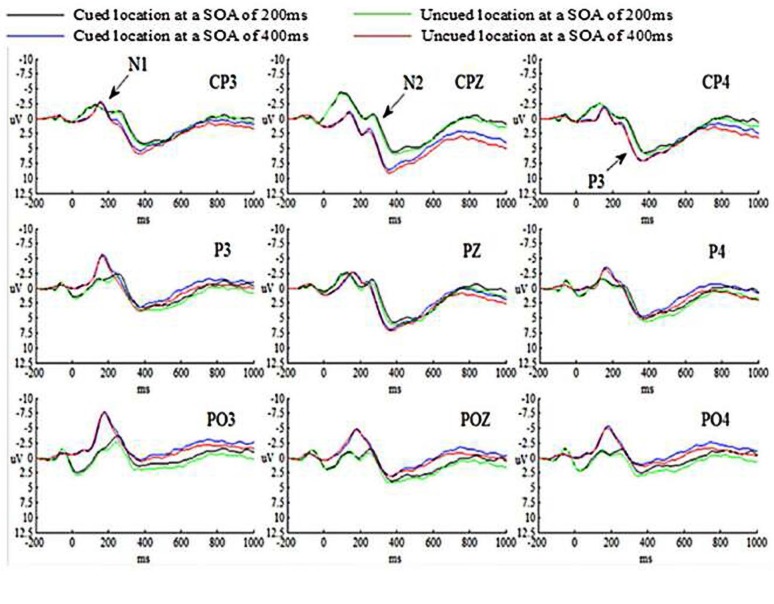
**Grand-averaged ERP waveforms for the emergency managerial experts in Experiment 2**.

The grand-average ERP waveforms for all conditions are presented in Figure 3.

##### N1 component

N1 amplitude was calculated as the mean amplitude. The time window for mean detection, the electrode sites and the experimental design for N1 in Experiment 2 were identical with those for N1 in Experiment 1.

The main effects of SOA, Cue validity and Hem were not significant (*p*s > 0.05). The main effect of Site was significant (*p* < 0.05), indicating that N1 amplitude evoked by targets at parietal cortex was smaller than those at parietal-occipital cortex, *p* = 0.039 (*p* < 0.05). And N1 amplitude evoked by targets at central-parietal cortex and parietal-occipital cortex was marginally significant, *p* = 0.053. But N1 amplitude evoked by targets at central-parietal cortex and parietal cortex was not significant, *p* = 0.833 (*p* > 0.05). All the interactions were not significant (*p*s > 0.05).

##### N2 component

N2 amplitude was calculated as the mean amplitude. The time window for mean detection, the electrode sites and the experimental design for N2 in Experiment 2 were identical with those for N1 in Experiment 1.

The main effect of SOA was significant (*p* < 0.05), suggesting that N2 amplitude evoked by targets at SOA of 200 ms was much larger than those at SOA of 400 ms. The main effects of Cue validity and Hem were not significant (*p*s > 0.05). The main effect of Site was significant (*p* < 0.01), indicating that N2 amplitude evoked by targets at central-parietal cortex was smaller than those at parietal cortex, *p* = 0.007 (*p* < 0.01), at parietal-occipital cortex, *p* = 0.000 (*p* < 0.01), and at parietal-occipital cortex (*p* < 0.001). The interaction between SOA and Cue validity was not significant (*p* > 0.05). However, the interaction of SOA × Cue validity × Hem was significant (*p* < 0.05). The simple effect on further test for comparison between cued and uncued condition showed that N2 amplitude evoked by targets at SOA of 200 ms in the right hemisphere was marginally significant, *p* = 0.059 (0.05 < *p* < 0.1), showing that N2 amplitude evoked by targets in cued trials was much larger than those in uncued trials at SOA of 200 ms, but not for the left and central hemisphere at SOA of 200 ms, *p* = 0.676 (*p* > 0.05), *p* = 0.538 (*p* > 0.05), respectively; no significant differences were found for the left, central and right hemispheres at SOA of 400 ms, *p* = 0.171 (*p* > 0.05), *p* = 0.457 (*p* > 0.05), *p* = 0.478 (*p* > 0.05), respectively. The interaction of SOA × Cue validity × Site was not significant (*p* > 0.05).

##### P3 component

P3 amplitude was calculated as the mean amplitude. The time window for mean detection, the electrode sites and the experimental design for P3 in experiment 2 were identical with those for N1 in Experiment 1.

The main effects of SOA, Cue validity, Hem and Site were not significant (*p*s > 0.05). The interaction between SOA and Cue validity was not significant (*p* > 0.05). However, the interaction of SOA × Cue validity × Hem was significant (*p* < 0.05). The simple effect on further test for comparison between cued and uncued condition showed that P3 amplitude evoked by targets at SOA of 200 ms in the right hemisphere was significant, *p* = 0.003 (*p* < 0.01), showing that P3 amplitude evoked by targets in cued trials was smaller than those in uncued trials at SOA of 200 ms, but not for the left and central hemispheres at SOA of 200 ms, *p* = 0.332 (*p* > 0.05), *p* = 0.104 (*p* > 0.05), respectively; no significant differences for the left, central and right hemispheres at SOA of 400 ms, *p* = 0.226 (*p* > 0.05), *p* = 0.662 (*p* > 0.05), *p* = 0.395 (*p* > 0.05), respectively. The interaction of SOA × Cue validity × Site was not significant (*p* > 0.05).

## Discussion

The aim of the present study was to explore the neuro-cognitive mechanism underlying IOR effects for emergency managerial experts and novices. The major findings were provided by behavioral data and ERPs data. In behavioral level, the IOR effects were robust at SOA of 200 ms and 400 ms for the novices and emergency managerial experts. The ERPs results suggested no IOR effects on N1, N2 and P3 amplitudes for the novices. However, the IOR effects on P3, possible on N2 amplitudes were found for the emergency managerial experts, showing a larger N2 and a smaller P3 evoked by targets in cued trials than those in uncued trials over the right parietal lobe and adjacent brain regions at SOA of 200 ms.

The behavioral data of experiments indicated that there were no differences of IOR effects for emergency pictures at SOA of 200 ms and 400 ms between two groups of participants. Therefore, behavioral data cannot provide indexes to distinct the IOR effects between the novices and emergency managerial experts. Except the IOR effects of behavioral data, the brain activities underlying IOR effects, which were reflected by the ERPs data in the present study, could provide some evidence to differentiate the IOR effects between the novices and experts.

For the novices, no IOR effects on N1, N2 and P3 were found, which were inconsistent with the studies by McDonald et al. (1999) and Wright et al. (2013), but consistent with studies by Hopfinger and Mangun (2001) and Zhang et al. (2012). The findings of ERPs for novices were inconsistent with behavioral findings.

For the emergency managerial experts, no IOR robust on N1 component was found. It was the same with the ERPs data for novice, and was similar to the prior findings by Hopfinger and Mangun (2001) and Satel et al. (2012). For the N2 component, the results indicated the IOR effect on N2 amplitude. Previous studies demonstrated that N2 component was closely associated with the information processing of the feature about stimuli in cue-target paradigm (Mangun and Buck, 1998), and that the familiar faces induced a higher N2 amplitude, i.e., the familiarity affected the N2 amplitude (Thomas and Weaver, 1975). Therefore the N2 reflects the recognition of familiar objects and extraneous stimuli, The present study found that N2 amplitude evoked by targets in cued trials was much larger than those in uncued trials at SOA of 200 ms. It could be explained that the activation level for N2 component was higher due to the familiar stimuli of emergency pictures under the processing for the characteristics of targets in cued location. Hence the more cognitive resources were mobilized by emergency managerial experts. This finding supported the IOR effect for emergency pictures at SOA of 200 ms in behavioral data for emergency managerial experts, and possibly, the dominant hemisphere of the IOR effect on N2 component was in the right parietal lobe and adjacent brain regions. For the P3 component, it’s amplitude evoked by targets in cued trials was smaller than those in uncued trials at SOA of 200 ms in the right hemisphere, indicating a robust of IOR with hemisphere dominance at SOA of 200 ms. The results were consistent with the behavioral results for emergency managerial experts. The similar findings have been observed by the previous studies of Eimer (1994) and Dai and Feng (2009). The P3 component is considered closely related to the brain of ERPs about attention (Kok, 2001; Kocer et al., 2008). Because IOR after attention is (voluntarily or reflexively) disengaged from the cued location, and attended objects (Zhou and Chen, 2008), the experts had more flexible attentional processing and higher visual search efficiency than novices. The findings of ERPs for the emergency managerial experts were consistent with behavioral findings, and demonstrated that there were IOR effects on N2 and P3 components, suggesting that the emergency managerial experts were specialized in attentional processing for emergency information due to their expertise.

In conclusion, based on the ERPs data, there is a robust IOR at SOA of 200 ms for the emergency managerial experts, not for the novices; P3 and possible N2 components in the right parietal lobe and adjacent brain regions are the biological indicators for IOR about emergency pictures for emergency managerial experts.

In summary, the purpose of the present study was to explore the IOR effects between emergency managerial experts and novices. In the present study, the expert-novice paradigm and emergency pictures were used. Due to the different experiences with emergency management between participants, the emergency managerial experts have more experiences with emergency stimuli than novices do. So the emergency pictures used by the study might cause increases in stress hormones (in particular cortisol) with pronounced cognitive and behavioral effects including memory consolidation and retrieval in the novices, which could might cause emotional reactions of the novices and influence their attentional processing. To the extent, a limitation of the study is to make sure whether the effects of stress hormones impact the experimental results. For this reason, a simple saliva cortisol measurement for participants could have helped to clarify this issue in further studies.

## Author Contributions

RC finished the article. LW and SW collected and analyzed the data.

## Conflict of Interest Statement

The authors declare that the research was conducted in the absence of any commercial or financial relationships that could be construed as a potential conflict of interest.

## References

[B2] Bryan L. Harter N. (1897). Studies in the physiology and psychology of the telegraphic language. Psychol. Rev. 4, 27–53. 10.1037/h0073806

[B3] Bryan L. Harter N. (1899). Studies on the telegraphic language: the acquisition of a hierarchy of habits. Psychol. Rev. 6, 345–375. 10.1037/h0073117

[B4] Chase W. G. Simon H. A. (1973a). Perception in chess. Cogn. Psychol. 4, 55–81. 10.1016/0010-0285(73)90004-2

[B5] Chase W. G. Simon H. A. (1973b). “The mind’s eye in chess,” in Visual Infor-Mation Processing, ed. Chase W. G. (New York, NY: Academic Press), 215–281.

[B6] Cheal M. L. Chastain G. (1999). Inhibition of return: support for generality of the phenomenon. J. Gen. Psychol. 126, 375–390. 10.1080/00221309909595372 10555866

[B7] Cheal M. Chastain G. Lyon D. R. (1998). Inhibition of return in visual identification tasks. Vis. Cogn. 5, 365–388. 10.1080/713756786

[B8] Chica A. B. Lupiáñez J. (2009). Effects of endogenous and exogenous attention on visual processing: an inhibition of return study. Brain Res. 1278, 75–85. 10.1016/j.brainres.2009.04.011 19374885

[B9] Dai Q. Feng Z. Z. (2009). Deficient inhibition of return for emotional faces in depression. Acta Psychol. Sinica 41, 1175–1188. 10.3724/sp.j.1041.2009.01175 19394388

[B10] Eimer M. (1994). An ERP study on visual spatial priming with peripheral onsets. Psychophysiology 31, 154–163. 10.1111/j.1469-8986.1994.tb01035.x 8153251

[B11] Eriksen C. W. Hoffman J. E. (1973). Extent of processing of noise elements during selective encoding from visual-displays. Percept. Psychophys. 14, 155–160. 10.3758/bf03198630

[B13] Folstein J. R. Van Petten C. V. (2008). Influence of cognitive control and mismatch on the N2 component of the ERP: a review. Psychophysiology 45, 152–170. 10.1111/j.1469-8986.2007.00602.x 17850238 PMC2365910

[B62] Francis L. Milliken B. (2003). Inhibition of return for the length of a line? Percept. Psychophys. 65, 1208–1221. 10.3758/BF03194846 14710956

[B14] Gobet F. Simon H. A. (2000). Five seconds or sixty? Presentation time in expert memory. Cogn. Sci. 24, 651–682. 10.1207/s15516709cog2404_4

[B12] Gouzoulis-Mayfrank E. Arnold S. Heekeren K. (2006). Deficient inhibition of return in schizophrenia—further evidence from an independent sample. Prog. Neuropsychopharmacol. Biol. Psychiatry 30, 42–49. 10.1016/j.pnpbp.2005.06.016 16014319

[B15] Gutiérrez-Domínguez F. J. Pazo-Álvarez P. Doallo S. Fuentes L. J. Lorenzo-López L. Amenedo E. (2014). Vertical asymmetries and inhibition of return: effects of spatial and non-spatial cueing on behavior and visual erps. Int. J. Psychophysiol. 91, 121–131. 10.1016/j.ijpsycho.2013.12.004 24342058

[B16] Hawkins H. L. Shafto M. G. Richardson K. (1988). Effects of target luminance and cue validity on the latency of visual detection. Percept. Psychophys. 44, 484–492. 10.3758/bf03210434 3226899

[B17] Hopfinger J. B. Mangun G. R. (2001). Tracking the influence of reflexive attention on sensory and cognitive processing. Cogn. Affect. Behav. Neurosci. 1, 56–65. 10.3758/cabn.1.1.56 12467103

[B18] Hopt J. M. Vogel E. K. Woodman G. F. Heinze H. J. Luck S. J. (2002). Localizing visual discrimination processes in time and space. J. Neurophysiol. 88, 2088–2095. 10.1152/jn.00860.2001 12364530

[B19] Jonides J. (1976). Voluntary vs. reflexive control of minds eyes movement. Bull. Psychon. Soc. 8, 243–244.

[B21] Klein R. M. (2000). Inhibition of return. Trends Cogn. Sci. 4, 138–147. 10.1016/S1364-6613(00)01452-2 10740278

[B60] Kocer B. Unal T. Nazliel B. Biyikli Z. Yesilbudak Z. Karakas S.. (2008). Evaluating sub-clinical cognitive dysfunction and event-related potentials (P300) in clinically isolated syndrome. Neurol. Sci. 29, 435–444. 10.1007/s10072-008-1020-4 19002651

[B23] Kok A. (2001). On the utility of P3 amplitude as a measure of processing capacity. Psychophysiology 38, 557–577. 10.1017/s0048577201990559 11352145

[B24] Krawczyk D. C. Boggan A. L. McClelland M. M. Bartlett J. C. (2011). The neural organization of perception in chess experts. Neurosci. Lett. 499, 64–69. 10.1016/j.neulet.2011.05.033 21635936

[B27] Lucia R. Ilaria P. Carlo U. (2004). Location and shape in inhibition of return. Psychol. Res. 68, 41–54. 10.1007/s00426-003-0136-7 12827352

[B28] Macinnes J. W. Klein R. M. (2003). Inhibition of return biases orienting du-ring the search of complex scenes. ScientificWorldJournal 3, 75–86. 10.1100/tsw.2003.03 12806122 PMC5974741

[B61] Mangun G. R. Buck L. A. (1998). Sustained visual-spatial attention produces costs and benefits in response time and evoked neural activity. Neuropsychologia 36, 189–200. 10.1016/S0028-3932(97)00123-1 9622184

[B29] Maylor E. A. (1985). “Facilitatory and inhibitory componenets of orienting in visual space,” in Attention and Performance XI, eds Posner M. I. Marin O. S. M. (Hillsdale, NJ: Lawrence Erlbaum Associates), 189–204.

[B30] McDonald J. J. Ward L. M. Kiehl K. A. (1999). An event-related brain po-tential study of inhibition of return. Percept. Psychophys. 61, 1411–1423. 10.3758/bf03206190 10572468

[B31] Mountcastle V. B. (1978). Brain mechanisms for directed attention. J. R. Soc. Med. 71, 14–28. 416210 10.1177/014107687807100105 PMC1436420

[B33] Posner M. I. (1980). Orienting of attention. Q. J. Exp. Psychol. 32, 3–25. 10.1080/00335558008248231 7367577

[B34] Posner M. I. Cohen Y. (1984). Components of visual orienting Attention and Performance X: Control of Language Processes, eds Bouma H. Bouwhuis D. G. (Hove, UK: Lawrence Erlbaum Associates Ltd), 531–556.

[B36] Pratt J. (1995). Inhibition of return in a discrimination task. Psychon. Bull. Rev. 2, 117–120. 10.3758/bf03214416 24203594

[B39] Prime D. J. Jolicoeur P. (2009). Response selection conflict contributes to inhibition of return. J. Cogn. Neurosci. 21, 991–999. 10.1162/jocn.2009.21105 18752398

[B38] Prime D. J. Ward L. M. (2004). Inhibition of return from stimulus to response. Psychol. Sci. 15, 272–276. 10.1111/j.0956-7976.2004.00665.x 15043647

[B40] Rafal R. D. Calabresi P. A. Brennan C. W. Sciolto T. K. (1989). Saccade preparation inhibits reorienting to recently attended locations. J. Exp. Psychol. Hum. Percept. Perform. 15, 673–685. 10.1037/0096-1523.15.4.673 2531204

[B41] Remington R. W. Johnston J. C. Yantis S. (1992). Involuntary attentionalcapture by abrupt onsets. Percept. Psychophys. 51, 279–290. 10.3758/bf03212254 1561053

[B43] Satel J. Wang Z. Hilchey M. D. Klein R. M. (2012). Examining the dissociation of retinotopic and spatiotopic inhibition of return with event-related potentials. Neurosci. Lett. 524, 40–44. 10.1016/j.neulet.2012.07.003 22801253

[B44] Sayegh L. Anthony W. P. Perrewe P. L. (2004). Managerial decision-making under crisis: the role of emotion an intuitive decision process. Hum. Res. Manage. Rev. 3, 179–199. 10.1016/j.hrmr.2004.05.002

[B45] Spence C. Lloyd D. McGlone F. Nichols M. E. R. Driver J. (2000). Inhibition of return is supramodal: a demonstration between all possible pairings of vision, touch, and audition. Exp. Brain Res. 134, 42–48. 10.1007/s002210000442 11026724

[B47] Thomas E. A. Weaver W. B. (1975). Cognitive processing and time perception. Percept. Psychophys. 17, 363–367. 10.3758/bf03199347

[B49] Vanselst M. Jolicoeur P. (1994). A solution to the effect of sample-size on outlier elimination. Q. J. Exp. Psychol. A 47, 631–650. 10.1080/14640749408401131

[B50] Vogel E. K. Luch S. J. (2000). The visual N1 components as an index of a discrimination process. Psychophysiology 37, 190–203. 10.1111/1469-8986.3720190 10731769

[B52] Wang Z. Klein R. M. (2010). Searching for inhibition of return in visual search: a review. Vision Res. 50, 220–228. 10.1016/j.visres.2009.11.013 19932128

[B54] Wright M. J. Gobet F. Chassy P. Ramchandani P. N. (2013). ERP to chess stimuli reveal expert-novice differences in the amplitudes of N2 and P3 components. Psychophysiology 50, 1023–1033. 10.1111/psyp.12084 23837745

[B55] Wurtz R. H. Goldberg M. E. Robinson D. L. (1980). Behavioral modulation of visual responses in the monkey: stimulus selection for attention and movement. Prog. Psychobiol. Psychol. 9, 43–83.

[B57] Zhang Y. Zhou X. Zhang M. (2012). Temporary inhibitory tagging at previously attended locations: evidence from event-related potentials. Psychophysiology 49, 1191–1199. 10.1111/j.1469-8986.2012.01412.x 22882160

[B59] Zhou X. Chen Q. (2008). Neural correlates of spatial and non-spatial inhibition of return (IOR) in attentional orienting. Neuropsychologia 46, 2766–2775. 10.1016/j.neuropsychologia.2008.05.017 18597795

